# A complex intragenic rearrangement of *ERCC8* in Chinese siblings with Cockayne syndrome

**DOI:** 10.1038/srep44271

**Published:** 2017-03-23

**Authors:** Hua Xie, Xiaoyan Li, Jiping Peng, Qian Chen, ZhiJie Gao, Xiaozhen Song, WeiYu Li, Jianqiu Xiao, Caihua Li, Ting Zhang, James F. Gusella, Jianmin Zhong, Xiaoli Chen

**Affiliations:** 1 Beijing Municipal Key Laboratory of Child Development and Nutriomics, Capital Institute of Pediatrics, Beijing, China; 2 Department of Neurology, Children’s Hospital of Jiangxi Province, Jiangxi, China; 3 Department of Neurology, Affiliated Children’s Hospital of Capital Institute of Pediatrics, Beijing, China; 4 State Key Laboratory of Genetic Engineering and Ministry of Education Key Laboratory of Contemporary Anthropology, School of Life Sciences, Fudan University, Shanghai, China; 5 Genesky Biotechnologies, ShangHai, China; 6 Molecular Neurogenetics Unit, Center for Human Genetic Research, Massachusetts General Hospital, Boston, MA, 02114, USA; 7 Department of Genetics, Harvard Medical School, Boston, MA, 02115, USA

## Abstract

Cockayne syndrome is an autosomal recessive disorder principally characterized by postnatal growth failure and progressive neurological dysfunction, due primarily to mutations in *ERCC6* and *ERCC8.* Here, we report our diagnostic experience for two patients in a Chinese family suspected on clinical grounds to have Cockayne syndrome. Using multiple molecular techniques, including whole exome sequencing, array comparative genomic hybridization and quantitative polymerase chain reaction, we identified compound heterozygosity for a maternal splicing variant (chr5:60195556, NM_000082:c.618-2A > G) and a paternal complex deletion/inversion/deletion rearrangement removing exon 4 of *ERCC8,* confirming the suspected pathogenesis in these two subjects. Microhomology (TAA and AGCT) at the breakpoints indicated that microhomology-mediated FoSTeS events were involved in this complex *ERCC8* rearrangement. This diagnostic experience illustrates the value of high-throughput genomic technologies combined with detailed phenotypic assessment in clinical genetic diagnosis.

Cockayne syndrome (CS; MIM# 133540, 216400), a rare autosomal recessive disorder first described by Cockayne in 1936^1^, involves multiple systems, and its major features include progressive growth failure, microcephaly, mental retardation, retinal pigmentary degeneration, sensorineural deafness, photosensitivity and premature death^2^. Different clinical subtypes of CS have been defined based upon severity and phenotypic heterogeneity^2^. For example, the classic form, CS type I, presents with developmental abnormalities in the first two years, and usually leads to death in the first or second decade. In contrast, CS type II is more severe with symptoms present at birth, while CS type III is a milder/later-onset form with normal growth and cognitive development. Due to its wide spectrum of symptoms, precise diagnosis of CS must rely on molecular genetic testing. CS cases may be caused by mutation in *ERCC8* (Cockayne syndrome A or CSA; 35% of cases) or *ERCC6* (Cockayne syndrome B or CSB; 65% of cases)^2^,^3^,^4^,^5^,^6^,^7^.

Here, we report our diagnostic experience for two clinically suspected CS patients in a Chinese family. Using multiple molecular techniques, including whole exome sequencing (WES), array comparative genomic hybridization (aCGH) and quantitative polymerase chain reaction (qPCR), we completed genetic diagnosis and counseling for this family, confirming CS due to compound *ERCC8* variants. Both siblings were compound heterozygotes for variants not reported previously: a maternal splicing variant (chr5:60195556, NM_000082:c.618-2A > G) and a paternal complex rearrangement removing exon 4 of *ERCC8* (chr5:60211534-60213756-60212086-60217114, hg19). Our report illustrates the diagnostic puzzle presented by some CS cases, and the advantage of approaching molecular diagnosis with high-throughput genomic techniques in the clinical genetics laboratory.

## Results

### Clinical report

The Capital Institute of Pediatrics Review Board approved this project. Written informed consent was obtained from the patients’ guardian/parent/next of kin for the publication of this report and any accompanying images.

**Figure 1:**
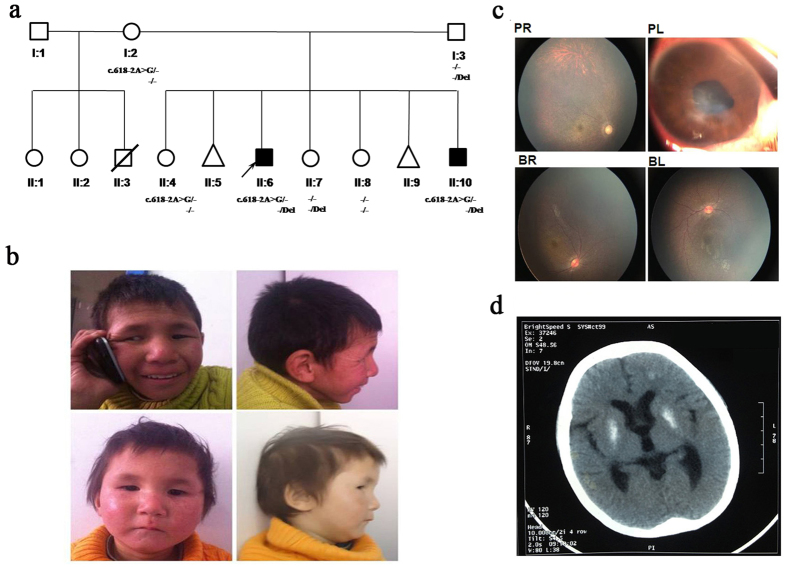
Pedigree and clinical characteristics of the two patients. (**a**) The family Pedigree. Black symbols denote affected individuals, and open symbols denote unaffected individuals. Genotypes are given for all family members who provided DNA. Del: the rearrangement removing exon 4 allele. -: the wild-type allele. (**b**) The facial features of the two patients. The top two photos show the proband (II:6); the bottom two photos show the affected sibling (II:10). (**c**) Fundus examination of the two patients. Figure PR and PL show optic nerve atrophy and retinitis pigmentosa in the right and left eye of the proband, respectively. Figure BR and BL show optic nerve atrophy in both eyes of the affected sibling. (**d**) The brain CT scan of the proband shows calcification of bilateral globus pallidus and subcortex of left frontal lobe, mild cerebral atrophy (widened sulcus and cleft, enlarged superatentorial ventricle) and cerebellar vermis dysplasia.

There are two affected boys in this large Chinese family. Their clinical phenotypes were evaluated by a neurologist, ophthalmologist, otolaryngologist, and a clinical geneticist. Their parents are nonconsanguineous and healthy. The mother had two marriages and ten pregnancies. During her first marriage she gave birth to one boy who died from pneumonia at one year of age and to two normal girls. Her second marriage produced three normal girls and the two affected boys in this study (Fig. 1a). Two abortions were also recorded during the second marriage.

The proband (II:6) is a 13 year old boy. He was severely hypotonic at birth and could not sit or stand until age four. At five years of age, he could sit steadily and stand with aid, and began to murmur simple words such as “baba” and “mama”. He was first referred to Jiangxi Children’s Hospital at six years old due to significant developmental delay and facial photosensitivity which failed to respond to any medical treatment. Subsequently, his parents pursued a medical diagnosis at many children’s hospitals. He was transferred to the affiliated hospital of the Capital Institute of Pediatrics, and given a detailed physical checkup and genetic counseling. At 10 years old, his height and weight was 93 cm and 12 kg, and head circumference was 42.5 cm; all were 3 standard deviation (SD) or more below the mean. He had dysmorphic facial features (Fig. 1b). The brainstem auditory evoked potential (BAEP) confirmed hearing loss, and the fundus examination showed optic nerve atrophy and retinitis pigmentosa (Fig. 1c). In addition, the brain CT scan showed calcification of bilateral globus pallidus, mild cerebral atrophy and cerebellar vermis agenesis (Fig. 1d). His clinical information is summarized in Table 1.

The affected brother (II:10) is three years old, with milder clinical features. At age of three, he can stand with aid, but can’t walk alone; he can say “baba” and “mama”, and can count from 1 to 10. Unlike his brother, he has bilateral cryptorchidism. His dysmorphic facial features and clinical information are summarized in Table 1. Fundus examination showed optic nerve atrophy (Fig. 1c). The brain CT scan showed cerebellar vermis dysplasia and decreased white matter volume (data not shown).

Based upon their major clinical and facial features (severe postnatal growth failure, neurologic dysfunction, intracranial calcifications), the two patients were thought to display CS. However, sequencing for *ERCC8/ERCC6* did not detect homozygous or compound heterozygous pathogenic variants although it did reveal a variant presumed to affect splicing in *ERCC8*. Karotyping and aCGH (Agilent SurePrint G3 4 × 180 K CGH+ SNP array) were performed for both patients, but no pathogenic genomic imbalance or uniparental disomy (UPD) was detected, so no precise genetic diagnosis could be given.

### Pathogenic variant and family co-segregation analysis

To complete genetic diagnosis and counseling for this family, we pursued WES for the two affected siblings (II:6 and II:10) in an attempt to identify the cause of pathogenesis for this as yet uncertain disorder.

An average of 55.80 million reads of DNA sequence were generated with average 49.37 fold coverage, and above 97.76% of the reads were aligned to hg19, providing sufficient depth to call single nucleotide polymorphisms (SNPs) and deletion/insertion (del/ins). Common variants reported in dbSNP138 or in the 1000 Genomes Project with minor allele frequency (MAF) ≥0.005 were excluded. The Exome Aggregation Consortium (ExAC) database was used to confirm the novelty of single nucleotide variants (SNVs). Given the similar characteristics of the two siblings, shared rare variants were collected and uploaded to Ingenuity Pathways Analysis (IPA) software for genotype-phenotype analysis. A total of 826 rare variants was shared between the two patients. To narrow these to a manageable number, we set key phenotypes including “photosensitivity”, “intellectual and developmental disabilities”, “dwarfism”, “hearing loss” and “retinitis pigmentosa” in the IPA system. According to the pedigree, autosomal recessive and X-linked recessive genetic disease were both possible models for inheritance in this family. Recently, the American College of Medical Genetics and Genomics (ACMG) developed standards and guidelines for the interpretation of sequence variants: the ACMG recommends five specific standard terminologies (pathogenic, likely pathogenic, uncertain significance, likely benign and benign) to describe any individual variant identified in genes known to cause Mendelian disorders^8^. We strictly followed ACMG guidelines to classify pathogenic/likely pathogenic variants from WES, with null variants (nonsense, frameshift, canonical splice sites, initiation codon, single exon or multiexon deletion) and predicted deleterious missense variants receiving preferential consideration. With this strategy, 23 heterozygous variants on autosomes and one variant on the X chromosome were chosen for co-segregation analysis using all family members (Supplementary Table S1).

**Figure 2:**
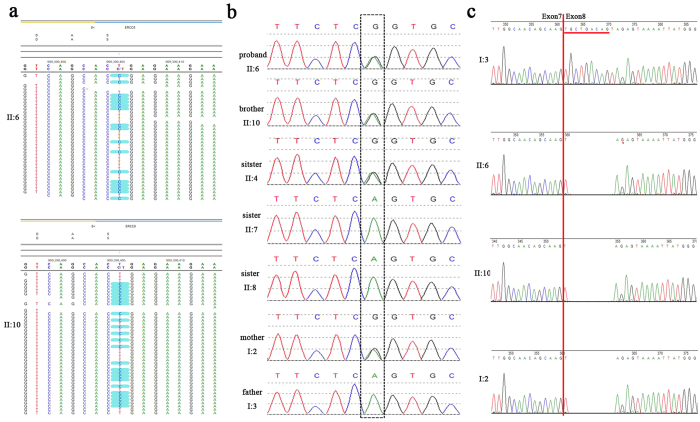
Identification and characterization of the c.618-2A > G splicing variant of *ERCC8*. (**a**) NextGENe views of the c.618-2A > G variant of two patients. (**b**) Sanger traces for PCR products show the c.618-2A > G variant in the family members, confirming it as a maternally inherited splice site variant. (**c**) Sanger traces for RT-PCR products show 9 bp (underlined in red) deleted from exon 8 in the two patients (II:6 and II:10) and their mother (I:2) due to the c.618-2 A > G splicing variant.

However, all but one of these candidate variants were excluded by co-segregation analysis: *ERCC8* c.618-2A > G (NM_000082), a potential splicing variant, was present in both siblings (viewed by NextGENe software in Fig. 2a). Subsequent Sanger sequencing in the family members confirmed that these two siblings and one normal sister (II:4) inherited this heterozygous variant from their mother, while the father and two other unaffected sisters have the reference sequence at this site (Fig. 2b).

### Splicing variant leads to deletion of three amino acid residues

To test whether the identified c.618-2A > G (NM_000082) variant does affect pre-mRNA splicing, we generated cDNA from RNA isolated from peripheral blood of the two patients and their parents. *ERCC8* reverse transcription polymerase chain reaction (RT-PCR) amplification products were cloned and sequenced. We found that this splicing variant resulted in absence in the mRNA of the first 9 bp (TGCTGACAG) of exon 8 (Fig. 2c), corresponding to loss of three amino acids (ADS, NP_000073) from position 207 to 209 within the fourth WD motif of the ERCC8 (CSA) protein.

### Complex *ERCC8* deletion/inversion/deletion rearrangement detected by qPCR and 1 M aCGH

**Figure 3:**
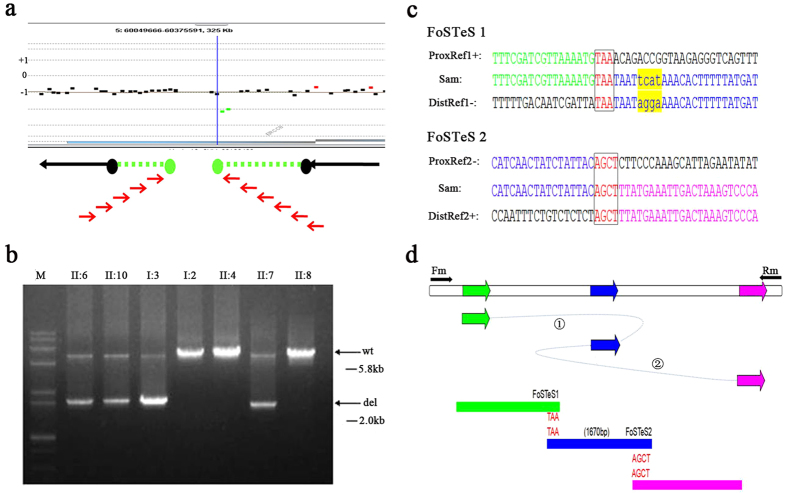
Identification and characterization of complex *ERCC8* exonic deletion. (**a**) The location of probes in the 1 M aCGH and the deletion-specific primers. The black rectangles indicate the locations of non-deleted probes while the green rectangles indicate deleted probes. The red arrows below the map area represent the deletion-specific primers which were designed to amplify the deletion breakpoints. (**b**) Long-range PCR shows that the heterozygous deletions were detected in two patients (II:6 and II:10), father (I:3) and one sister (II:7). The 5.8 kb band is the wild-type allele (wt) and the 2.0 kb band is the deletion allele (del). (**c**) Two FoSTeS events are revealed by sequence analysis of the breakpoints. Two microhomologies (TAA and AGCT, respectively) are found at four breakpoint sites. Four base del/ins bases (tcat, yellow) were identified near one junction. (**d**) The putative rearrangement model of complex *ERCC8* exonic deletion (not to scale) illustrates that two putative FoSTeS events occurred at four genomic rearrangement sites, resulting in deletion/inversion/deletion in the *ERCC8* region. Fm and Rm were used to amplify the 2.0 kb joint product.

We hypothesized that perhaps an undetected intragenic deletion in *ERCC8* combined with the splicing variant to generate the CS phenotype. Accordingly, both exon-targeted qPCR and high density 1 M aCGH were performed to test this possibility. Using primers targeted on each exon of *ERCC8*, we detected a possible deletion covering exon 4 in the affected siblings (Supplementary Fig. S1). Meanwhile, 1 M aCGH conducted for the proband detected an abnormal log ratio for two probes (chr5:60212688-60219701, 7 kb, green dots in Fig. 3a) in *ERCC8* suggesting the possibility of a small intragenic deletion. Long-range PCR with a series of deletion-specific primers (red arrowheads in Fig. 3a, Fm and Rm primers in Supplementary Table S2) was then performed in all family members to confirm and map this intragenic deletion. Fragment size analysis indicated deletion of 3.8 kb in the two affected patients (Fm5 and Rm4 primers in Supplementary Table S2), the father (I:3) and one sister (II:7), but not in the mother and the two other sisters (II:4 and II:8) (Fig. 3b).

To confirm the breakpoints of this intragenic *ERCC8* deletion, we purified the deletion-specific PCR product from an agarose gel and sequenced it (using the Fsq, Rsq, Fm5, Rm4, primers in Supplementary Table S2). These sequencing data revealed a complex deletion/inversion/deletion rearrangement (chr5:60211534-60217114) in which two distinct deletions (chr5:60211534-60213756 which removes exon 4 and chr5:60212086-60217114) flank a 1670 bp inverted segment (chr5:60212086-60213756). The sequence characteristics surrounding the four breakpoints revealed microhomologies (TAA on chr5:60211534/60213756; AGCT on chr5:60212086/60217114. see Fig. 3c,d) as potential factors contributing to the *ERCC8* rearrangement. We also performed the cDNA sequencing for all available family members, and confirmed the skipping of exon 4 in the proband, sibling and father (Supplementary Fig. S2).

## Discussion

*ERCC8* (encoding CSA protein) is located on chromosome 5q12.1 and encodes a 44-kDa protein of 396 amino acids with seven predicted WD-40 repeat motifs^5^,^9^. CSA interacts with CSB (the product of *ERCC6*) to recruit other repair factors (XAB2, HMGN1, TFIIS) to the repair site during repair of UV-generated DNA damage^10^,^11^,^12^,^13^. Mutations in *ERCC8* account for about 35% of the CS cases and comprise nonsense, missense, frameshift and splicing mutations, along with dosage imbalance due to small insertions and deletions^7^,^14^. In the current study, we investigated two boys characterized by growth failure, neurological impairment, microcephaly, short statue, hearing and insight loss, and photosensitivity, the characteristics of classical CS (CS type I). However, the genetic diagnosis was not given to the family until the proband was 13 years old. We performed multiple genetic testing modalities for the affected siblings to finally achieve a precise molecular diagnosis for this family. The affected siblings carry a splicing variant and a complex exonic deletion of *ERCC8*, which were inherited from the mother and the father, respectively. Both variants were interpreted as “pathogenic” in a recessive mode, according to the guidelines of ACMG based upon the following criteria: absent in population databases, predicted as null/deleterious variants, co-segregating with disease in multiple affected family members, presence in *trans*^8^. The maternal splicing variant (c.618-2A > G) leads to an in-frame deletion of 9 bp in exon 8, resulting in loss of three amino acids (ADS) within the fourth WD motif of CSA. At this same splice site, a different variant, c.618–1G > A, has been reported repeatedly in Caucasian patients, and the transcript analysis has confirmed that it also produces mis-splicing that removes the same amino acids (ADS) within the same WD motif^7^,^15^. We reviewed CS case reports and found that all causative missense mutations of *ERCC8* identified in CS patients are located in the WD repeats^7^,^16^, implying the importance of WD motifs for building beta propeller structures and protein–protein interactions. *In vitro* functional analysis has also indicated that missense mutation (A205P) in the fourth WD motif affects its interaction with other proteins (DDB1)^17^. Beside SNVs, exonic deletions account for 16.7% (7/42) of *ERCC8* mutations in CS^7^. Examples include a deletion containing exon 1 and upstream regulatory sequences in a CS patient reported by Ren *et al*.^18^ and double deletions in a CS patient composed of a paternally-inherited deletion covering exon 4 and a maternal whole gene deletion of *ERCC8*^14^. In our patients, the deletion of exon 4 was so small that it was easily missed during routine aCGH testing due to few probes covering each exon of the *ERCC8* region on the particular arrays used. This initiated a long diagnostic odyssey for this family.

With the dramatic improvements in genomic technologies and the significantly decreased price for sequencing, high-throughput whole genomic testing has been accepted as the most comprehensive and valuable testing approach for clinical diagnosis laboratory^19^,^20^,^21^. In general, high-throughput whole genomic testing includes whole genomic aCGH and WES. The former can detect genomic imbalances more effectively than FISH or karyotyping^22^,^23^, and the latter can detect any SNVs and del/ins in coding sequences across the genome. Compared with candidate gene or gene panel sequencing, WES provides an unbiased approach to affordably screen a patient’s entire exome to establish the genetic basis of disease. These high throughout genomic technologies have increased the diagnostic yield among individuals with unexplained developmental disorders, and rapidly revolutionized molecular diagnostics strategy, enabling physicians and patients to move to more accurate diagnostics and appropriate treatment (precision medicine). For patients with unknown unexplained autism spectrum disorder (ASD), the clinical utility of chromosomal microarray analysis (CMA) has dramatically increased the rate of diagnosis from 2.23% to 7%^24^. Recently, the Scherer group combined CMA and WES assays to study the genomic basis for a heterogeneous sample of ASD patients; they found that the combined molecular diagnostic yield was 15.8%, higher than the diagnostic yield of WES (8.4%) or CMA (9.3%) alone^25^. However, these high-throughput genomic testing approaches cannot handle all diagnostic puzzles if the geneticist does not fully integrate the information from these different techniques with accurate and complete clinical data. The identification of precise/unique symptoms is a key step in phenotype-genotype interpretation during WES. When we initially performed data analysis in the IPA system, we used the symptoms “intellectual and developmental disabilities”, “dwarfism”, and “hearing loss” but failed to identify the mode of pathogenesis because these symptoms are seen in many genetic/genomic disorders. In the second round of interpretation, we used two more specific symptoms (photosensitivity and retinitis pigmentosa) for data analysis, reducing the candidate variant pool sufficiently to permit testing of co-segregation in family members. Our results indicated the value of repeated and detailed review of the detailed phenotype of the patient to prioritize and ultimately identify causative pathogenic variants in clinical WES testing.

Notably, deletion of *ERCC8* has been identified in the normal population, as evidenced by the Database of Genomic Variant track in the UCSC Genome Browser, indicating no evidence of a clinical phenotype caused by haploinsufficiency. We compared the frequency of intragenic *ERCC8* deletions from one large control cohort (dgv9811n54, dgv9810n54)^26^ and one CS patient cohort (Laugel’s study)^7^. *ERCC8* deletion was significantly more frequent in CS patients than in controls (7/42 vs. 12/8329, p < 0.001, two-tailed Fisher’s exact test). This emphasized that, while *ERCC8* deletion is not recognized as a pathogenic CNV in the normal population, it is involved in the pathogenesis of CS when it co-occurs in *trans* with another pathogenic *ERCC8* variant. While in the process of submitting this paper, we identified another unrelated CS patient carrying exact same exonic deletion of *ERCC8* as the two patients in this study (data not shown), arguing that otherwise benign *ERCC8* deletions in the general population represent a risk for causing CS in a recessive model of inheritance.

Copy number variants (CNVs) are generated by a variety of genomic rearrangements^27^,^28^,^29^,^30^,^31^,^32^,^33^,^34^. Non-allelic homologous recombination (NAHR) between low-copy repeats can cause recurrent rearrangements and lead to genomic disorders, such as DiGeorge syndrome and Williams-Beuren syndrome^35^. Non-homologous end-joining (NHEJ) is another mechanism contributing to chromosomal abnormality via the formation and repair of DNA double-strand breaks (DSBs), and is one of the key mechanisms underlying non-recurrent rearrangements^36^. DNA replication fork stalling and template switching (FoSTeS) is a recently identified mechanism for non-recurrent and especially complex rearrangements due to faulty DNA replication. A short stretch of microhomology is a key feature of the FoSTeS process. The FoSTeS molecular mechanistic details have been provided in the microhomology-mediated break-induced replication (MMBIR) model. The DNA replication FoSTeS/ MMBIR mechanism can generate complex genomic, genic and exonic rearrangements in humans^37^. FoSTeS had been implicated in many pathogenic genomic rearrangements, such as non-recurrent intragenic *NRXN1* deletion^38^ and duplication/deletion in *PLP1* associated with Pelizaeus-Merzbacher disease (PMD)^39^. In the current study, we mapped this complex *ERCC8* intragenic rearrangement (deletion/inversion/deletion) at the nucleotide level and found microhomologies (TAA and AGCT) around the breakpoint sites supporting the view that two FoSTeS events were involved in generating the *ERCC8* complex rearrangement.

In summary, we describe the clinical and genetic characterization of two affected boys with CS type I in a Chinese family. Following the guidelines for the interpretation of sequence variants of the ACMG, we identified compound heterozygosity, including a paternal intragenic rearrangement and a maternal splicing variant in *ERCC8*, as the cause of CS pathogenesis in these individuals. In the clinical diagnosis of many rare neurological diseases, combined utilization of careful clinical phenotyping with high-throughput genomic technologies, such as WES and aCGH, can increase the diagnostic yield and provide much-needed answers for families faced with these disorders.

## Methods

### Ethics statement

This study was performed in accordance with the Declaration of Helsinki and approved by the ethics committee of Capital Institute of Pediatrics (SHERLL 2015069). Written informed consent was obtained from the patients’ guardian/parent/next of kin for the publication of this clinical information and any accompanying images.

### aCGH

Genomic DNAs of the proband and all available family members were extracted from peripheral blood using the QIAamp DNA Blood Mini Kit (Qiagen, Hilden, Germany) according to the manufacturer’s instructions. Two aCGH (4 × 180 K and 1 M) (Agilent Technologies, Palo Alto, CA, USA) were used to detect genomic imbalance according to previously published methods^40^. aCGH data were analyzed via DNA CytoGenomics software (Agilent Technologies, Palo Alto, CA, USA).

### Whole exome sequencing and variant analysis

Genomic DNAs of the two patients were fragmented by Covaris S2 (Covaris, Massachusetts, USA) to 200–300 bp. The paired-end libraries were prepared following the Illumina protocol. Whole exome sequences were captured from the genomic DNA using Agilent SureSelect V5 Enrichment kit (Agilent, Cedar Creek, TX). The exon-enriched DNA libraries were sequenced by 100 bp paired-end reads on a Hiseq2000 sequencer (Illumina, San Diego, California). Raw image files were processed by the Illumina Pipeline for base calling using default parameters.

After image analysis and base calling was conducted using the Illumina Pipeline, raw data were transfer into fastq form and filtered to generate “clean reads” by removing adapters and low quality reads (Q20). Sequencing reads were mapped to the reference human genome version hg19 (200902 release, http://genome.ucsc.edu/) and variants were visualized and analyzed by NextGENe software version 2.1.1.1 (SoftGenetics, StateCollege, PA) and Ingenuity Variant Analysis software (http://www.ingenuity.com).

### qPCR

To test whether the affected patients (II:6, II:10) had the *ERCC8* intragenic deletion, qPCR was performed using the 7500 Fast Real-Time PCR System with *GAPDH* as internal reference gene for each sample. Two unrelated testing samples without *ERCC8* deletion (validated by 244k aCGH beforehand) were used as controls. The qPCR was carried out in the presence of SYBR Green, measuring the fluorescence signal produced by the binding of SYBR Green to the studied amplicons compared with the control. The qPCR primers for reference and target genes are in Supplementary Table S3.

### Long-range PCR and breakpoint mapping on the junction sequence

Long-range PCR was carried out to amplify the truncated fragment covering exon 4 of *ERCC8* and map out the characteristics of breakpoints (Platinum PCR SuperMix High Fidelity kit, Invitrogen, Carlsbad, CA). Using a series of primers around the approximate breakpoints (Supplementary Table S2), the junction fragments were amplified successfully and were visualized on a 1% agarose gel. The truncated fragment was purified from the gel (QIAquick gel extraction kit, Qiagen, Valencia, CA) and sequenced (ABI 3700).

### RT-PCR, Cloning and Sequencing

Total RNA was isolated from fresh peripheral blood using the RNeasy mini kit (Qiagen, Hilden, Germany) and was reverse transcribed with ProtoScript^®^ First Strand cDNASynthesis Kit (New England Biolabs Inc., MA, USA). PCR amplification of cDNA including spanning exon 3-12 was performed using the primer pair 5′-CACATGTAAAGCAGTGTGTTCC-3′and 5′-GCATTTCATGTTTAAGCCAGATT-3′, visualized on a 1% agarose gel, purified using QIAquick PCR Purification Kit (Qiagen, Hilden, Germany), and then cloned into a pUC19 vector for Sanger sequencing following the manufacturer’s protocol. A mixture of DNA from 10 normal children was used as the control.

## Additional Information

**How to cite this article**: Xie, H. *et al*. A complex intragenic rearrangement of *ERCC8* in Chinese siblings with Cockayne syndrome. *Sci. Rep.* **7**, 44271; doi: 10.1038/srep44271 (2017).

**Publisher's note:** Springer Nature remains neutral with regard to jurisdictional claims in published maps and institutional affiliations.

## Supplementary Material

Supplementary Information

## Figures and Tables

**Table 1:** Clinical information for the two patients.

	II:6	II:10
Age	13 years old	3 years old
Gender	M	M
Growth Parameters		
Height	99 cm (<3SD^a^)	80.5 cm (<3SD^a^)
Head circumference	43.5 cm (<3SD^a^)	43.5 cm (<3SD^a^)
Facial features		
nasal base breadth	+	+
protruding ears	+	+
micrognathia	+	+
poorly aligned teeth	+	+
Mental retardation	+	−
Growth delay	+	+
Hypotonia	+	+
Visionloss	+	+
Hearing loss	+	+
Photosensitivity	+, obviously	+, not obviously
Karyotype analysis	Normal	Normal

^a^SD standard deviation (http://www.who.int/childgrowth/standards/en/).
