# Supplementary material for: A complex intragenic rearrangement of ERCC8 in Chinese siblings with Cockayne syndrome
Source: Sci Rep. 2017 Mar 23;7:44271. doi: 10.1038/srep44271 (PMC5363064; doi:10.1038/srep44271)

A complex intragenic rearrangement of *ERCC8* in Chinese siblings with Cockayne syndrome

Hua Xie, Xiaoyan Li, Jiping Peng, Qian Chen, Zhijie Gao, Xiaozhen Song,Weiyu Li, Jianqiu Xiao, Caihua Li, Ting Zhang, James F Gusella, Jianmin Zhong, Xiaoli Chen

Table S1. Shared SNVs in two sibs with key phenotypes (retinitis pigmentosa and photosensitivity)

Gene Symbol	Position	Transcript ID	Transcript Variant	Protein Variant	Translation Impact	SIFT Prediction	PolyPhen2 Prediction	1000 Genomes	ExAC
OR2W3	chr1:248059126	NM_001001957.2	c.238C>T	p.Q80*	stop gain				0
COL5A2	chr2:189916123	NM_000393.3	c.2854C>T	p.R952C	missense	Damaging	Probably Damaging	0.06	0.02
ZNF595	chr4:59435	NM_182524.3	c.116A>C *	p.N39T	missense	Damaging	Probably Damaging		
ZNF595	chr4:86531	NM_182524.3	c.588G>T	p.W196C	missense		Possibly Damaging		
PPAT	chr4:57261610	NM_002703.4	c.1462C>T	p.Q488*	stop gain				
FAT1	chr4:187538271	NM_005245.3	c.8963A>T	p.K2988I	missense	Damaging	Benign	0.18	0.13
DNAH5	chr5:13719123	NM_001369.2	c.12367C>T	p.H4123Y	missense	Damaging	Possibly Damaging	0.08	0.07
ERCC8	chr5:60195556	NM_000082.3	c.618-2A>G						
F2RL1	chr5:76129192	NM_005242.4	c.760C>T	p.P254S	missense	Damaging	Probably Damaging		
COL11A2	chr6:33154514	NM_001163771.1	c.688G>T	p.G230W	missense	Damaging	Possibly Damaging	0.18	0.06
NT5DC1	chr6:116442132	NM_152729.2	c.529+3024C >T	p.G383R	missense		Probably Damaging	0.18	0.06
LAMB1	chr7:107569960	NM_002291.2	c.4642C>T	p.R1548C	missense	Damaging	Possibly Damaging	0.06	0.04
GOT1L1	chr8:37796274	NM_152413.2	c.241C>T	p.Q81*	stop gain			0.04	0.03
TNFRSF11B	chr8:119945260	NM_002546.3	c.310G>A	p.V104M	missense		Possibly Damaging	0.2	0.08
FBXO18	chr10:5979079	NM_001258452	c.2797A>T	p.R933W	missense	Damaging	Possibly Damaging		
PCDH15	chr10:55568720	NM_001142769.1	c.5113_5115 del	p.1705_1705 del	in-frame				0
CDH23	chr10:73573061	NM_022124.5	c.9694C>T	p.R3232W	missense		Probably Damaging		
NRG3	chr10:84738707	NM_001165973.1	c.751A>C	p.S251R	missense	Damaging	Possibly Damaging	0.44	0.23
FREM2	chr13:39264141	NM_207361.5	c.2660G>C	p.G887A	missense		Probably Damaging		0
SULT1A2	chr16:28603710	NM_001054.3	c.649G>T	p.E217*	stop gain			0.36	0.11
CDH15	chr16:89245912	NM_004933.2	c.131G>A	p.R44Q	missense	Damaging	Possibly Damaging	0.08	0.02
PER1	chr17:8052613	NM_002616.2	c.869A>G	p.D290G	missense	Damaging	Probably Damaging	0.02	0.01
CYP4F3	chr19:15760046	NM_000896.2	c.602A>G	p.D201G	missense	Damaging	Probably Damaging	0.12	0.06
PLXNA3	chrX:153688924	NM_017514.4	c.401C>T	p.P134L	missense	Damaging	Probably Damaging	0.03	0

Table S2. The primers for the Long-range PCR and breakpoint-specific PCR assay

Primer	Sequence (5' to 3')
Fm1	GGCAGCCCACATTCCAATTT
Fm2	TGCCAGGCTGAGTTTAGACA
Fm3	GAGGTTTGGACATTTGCCCA
Fm4	ACTCTAGCTTGGGGAACAGG
Fm5	CTTCCAGAGACCACCCACAT
Fm6	CAATGGAGGTGCTGGTTTGG
Rm1	CAGGAGGGAGGAAGAAAGGT
Rm2	CTGCGAAGACAGGAACCATG
Rm3	CACTCACCCCTGTCTTGTCT
Rm4	GGGAGAATGGGCACTGAGAA
Rm5	GCGTGTTGAAAACCGAAACC
Rm6	TGTTTGAGTGGCCCATGGTA
Rm7	AGGCAAAGAAGATCCAGCAT
Fsq1	TGGCATCTACAGTCTTGGCT
Rsq1	CCAAACCAGCACCTCCATTG
Fsq2	CATGTTCAGTTGGGACCATGT
Rsq2	GGATTAAGGGGATCACAAGCC
Rsq3	GCTTTGAAGATGGATTAAGGGGA
Rsq4	AGCCAAGACTGTAGATGCCA
Rsq5	ACCAAGCTATGCGAGACCAT
Rsq6	TTGTTGGCTCTGGTGTTTCG
Fsq3	ACATAGACATCTGGGAGCGG
Fsq4	CTGGTGTAGGCCCATGAGAT

* Fm and Rm represent the deletion-specific primers for breakpoint mapping, and Fsq and Rsq represent the breakpoint-specific primers for sequencing.

Table S3. The primers for qPCR assay

Primer	Sequence (5'to3')
ERCC8-1-F	TGAGGACACGATATGCTGGG
ERCC8-1-R	TACCTCCGTGTTGACTCTGC
ERCC8-2-F	TAGTAACGGCGCTCACCATT
ERCC8-2-R	AACCCCAGCATATCGTGTCC
ERCC8-3-F	AACATCGAACCTACCAAATGTTG
ERCC8-3-R	TCTAGCACCTAAAAGCCCTCT
ERCC8-4-F	GCAACTTAAGGATTGACGGTGA
ERCC8-4-R	CCATTGCTCTTTGTTGTGATGTG
ERCC8-5-F	AAGAAACCAAGGCAAAAGAGC
ERCC8-5-R	GCCAGAAAGCCCAAAGTCAA
ERCC8-6-F	CAGTGTGTTCCATTGGCAGG
ERCC8-6-R	CCATTCGCTTGACCCATTCA
ERCC8-7-F	TATCAGGTGGTTCAGATGGTGT
ERCC8-7-R	TGGAACACACTGCTTTACATGT
ERCC8-8-F	GATGTGAAAGCAGTACAGTGGT
ERCC8-8-R	GAGGTTTGGACATTTGCCCA
ERCC8-9-F	CATGTGATCTCGGTTTGGCC
ERCC8-9-R	AGGATGATCTCTACAAAACAGCA
ERCC8-10-F	TGTCTTTTGATTACCAGACTGCA
ERCC8-10-R	AACAGTGCTTGGTGGAGACT
ERCC8-11-F	AGTCTGGATCCTGTTCTCACA
ERCC8-11-R	TCAACAACCAGCACAAAGTACA
ERCC8-12-F	TTGGCCTCACTTTCTTCAGAA
ERCC8-12-R	TCTTGTCTGTGACCTGCAAA
ERCC8-13-F	AATTATGGGATGTGAGAAGAGCA
ERCC8-14-R	GCGTTCTTCCTTACCTGATTCA
ERCC8-15-F	GGGTGAGGGGTACAGTCATT
ERCC8-15-R	TCCTGATGCTCTTCTCACATC
ERCC8-16-F	CAACCCACCCTTACCCACTA
ERCC8-16-R	TACTACGCTTTGCACTCCCA
ERCC8-17-F	ACTGTTGACTGCTGTGTATTTCA
ERCC8-17-R	AACTGGTCTGGCAAGCTAGC
ERCC8-18-F	CATTGGCCCTTGTTCTGTCA
ERCC8-18-R	ACACAACAAAGCCTAGGAGCAA
ERCC8-19-F	ACTGCAACATTCTGGCTTGG
ERCC8-19-R	AGAAGTCACTGTACCATTTGTGA
ERCC8-20-F	TCAGCTCTTGATCCACAGTATCA
ERCC8-20-R	TGGAACCCAAGCCAGAATGT
ERCC8-21-F	CCTCAGTTGTGACCTTCAGC
ERCC8-21-R	TGGAAACGAGCAGGCTGATA
ERCC8-22-F	GATGTTGTGGCCTGGGAAAG
ERCC8-22-R	GCTGATCCTCTGAGACTGCA

Figure S1. Exon-targeted qPCR results show exon 4 deletion of *ERCC8* in the two patients

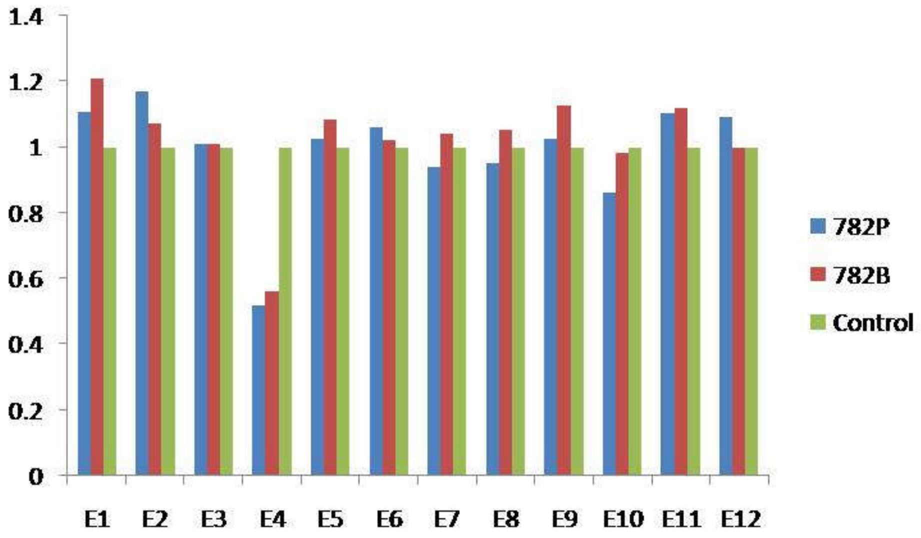

Figure S2. RT-PCR sequence results show exon 4 deletion of *ERCC8* in the proband, sibling and father

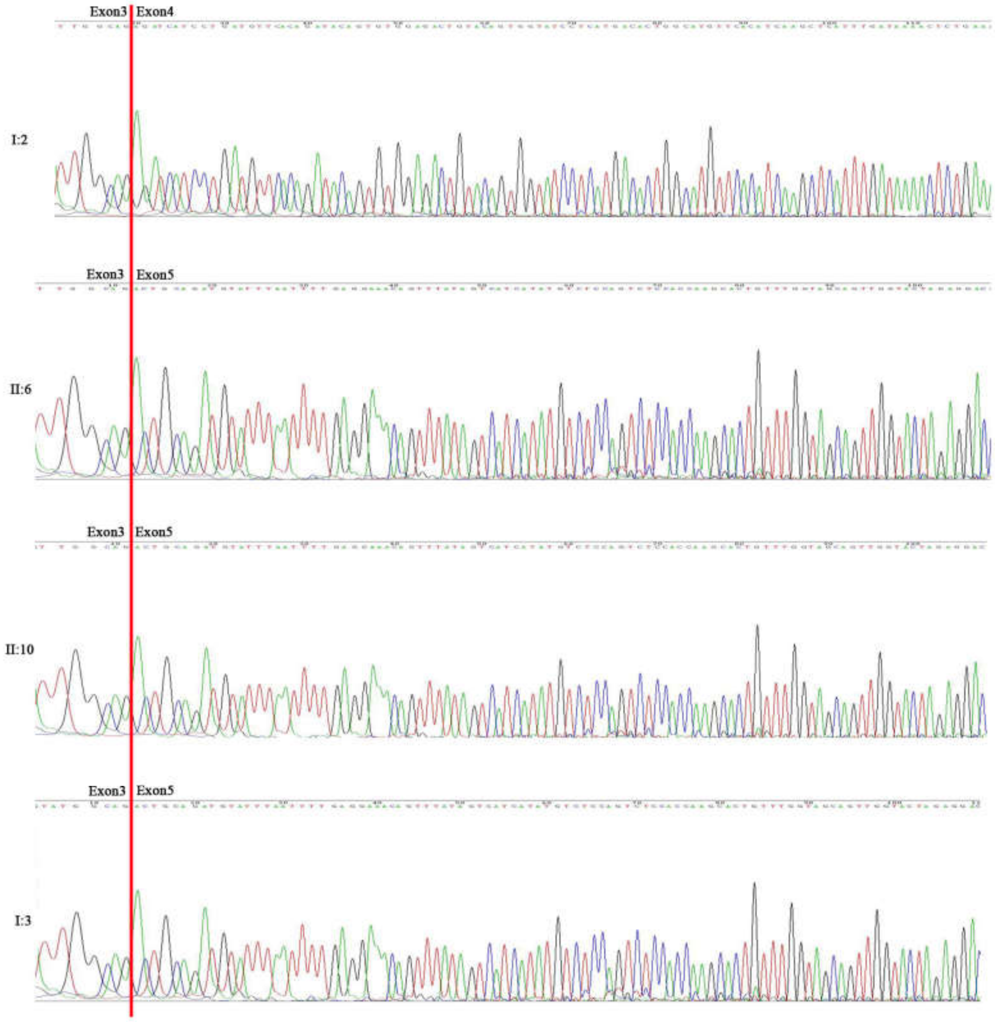

Supplement: Supplementary Information [file srep44271-s1.pdf]
